# Evidence for divergent selection between the molecular forms of *Anopheles gambiae*: role of predation

**DOI:** 10.1186/1471-2148-8-5

**Published:** 2008-01-11

**Authors:** Abdoulaye Diabaté, Roch K Dabiré, Kyle Heidenberger, Jacob Crawford, William O Lamp, Lauren E Culler, Tovi Lehmann

**Affiliations:** 1 Laboratory of Malaria and Vector Research, NIAID, National Institute of Health, 12735 Twinbrook Parkway, Room 2W13A, Rockville MD 20852 USA; 2 IRSS/Centre Muraz, Laboratoire de Parasitologie/Entomologie BP 390 Bobo Dioulasso, Burkina Faso; 3 Department of Entomology University of Maryland College Park, MD 20742-4454 USA

## Abstract

**Background:**

The molecular forms of *Anopheles gambiae *are undergoing speciation. They are characterized by a strong assortative mating and they display partial habitat segregation. The M form is mostly found in flooded/irrigated areas whereas the S form dominates in the surrounding areas, but the ecological factors that shape this habitat segregation are not known. Resource competition has been demonstrated between species undergoing divergent selection, but resource competition is not the only factor that can lead to divergence.

**Results:**

In a field experiment using transplantation of first instar larvae, we evaluated the role of larval predators in mediating habitat segregation between the forms. We found a significant difference in the ability of the molecular forms to exploit the different larval sites conditioned on the presence of predators. In absence of predation, the molecular forms outcompeted each other in their respective natural habitats however, the developmental success of the M form was significantly higher than that of the S form in both habitats under predator pressure.

**Conclusion:**

Our results provide the first empirical evidence for specific adaptive differences between the molecular forms and stress the role of larval predation as one of the mechanisms contributing to their divergence.

## Background

Divergent natural selection between populations inhabiting different ecological environments has long been thought to be a major cause of speciation [1]. Whereas there are now more examples of this process, the underlining mechanisms have been rarely examined [2]. Resource competition has been demonstrated between species undergoing divergent selection in some cases including seed-eating rodents [3], gerbil species [4], and sticklebacks [5]. The role of other mechanisms, such as predation, has long been discussed but remained controversial [6]. Part of the controversy stems from the fact that predation is extremely difficult to study in the field. Using a transplantation experiment, we demonstrated that the molecular forms of *Anopheles gambiae *differ in their ability to exploit different larval habitats and we provide evidence that larval predation contributes to the divergent selection involved.

The African malaria mosquito, *An. gambiae *is undergoing speciation [7,8], and yet the evolutionary forces that have been separating subpopulations of this species are not known. Coluzzi and others have hypothesized that human-made modifications of the African environment have created new ecological niches in marginal habitats and, thus, new opportunities for specialisation for this mosquito species [7,9,10]. Five chromosomal forms were identified in *An. gambiae*: Forest, Savanna, Bamako, Mopti, and Bissau [7,8]. Subsequent studies revealed two "molecular" forms (M and S) characterized by fixed nucleotide differences in the intergenic spacer of the ribosomal DNA [11]. The incomplete correspondence between the chromosomal and the molecular forms [12] complicated their taxonomic resolution as were the findings of low genetic differentiation between the forms in all genomic regions except the inversion and near the rDNA [13-17]. Although a strong deficit of M/S hybrids is observed in the field [12], the forms interbreed in the laboratory and their offspring are viable and fertile [18]. Recent studies provide support for genetic differentiation between the forms in a few limited spots of the genome [19,20]. These authors suggested that the genes in these spots called 'speciation islands' are responsible for the premating reproduction barrier [21,22] and the ecological adaptation of the forms to specific environments.

Ecological studies revealed a strong pattern of spatial and temporal segregation between the molecular forms, with the M form associated with drier conditions than the S form [8,23,24]. Most segregation occurred between rice cultivation areas, dominated by the M form, and their surrounding areas that are dominated by the S form, suggesting that segregation between the forms is related to the larval habitats. A previous study that evaluated differences in the capacity of larvae of the molecular forms to exploit rice fields and puddles in the absence of predators found no evidence for such adaptation when the forms were separated [25]. However, when cohabiting the same site the S form outcompeted the M form [25]. These findings prompted us to evaluate the mediating effect of larval predation on the development success of the molecular forms in these habitats.

Habitat selection is among the most important decisions that a female mosquito makes, since it determines the fate of her offspring. Factors that strongly affect the prospects of mosquito larvae include desiccation, nutrients, competition, and predation. Several studies have stressed the role of predators in controlling mosquito population sizes in the field. An overall estimate of 94% mortality of larvae due to predation was reported highlighting the huge selection pressure exerted on mosquito populations [26,27]. Theory suggests that the strength of divergent selection is mainly determined by the rate at which interspecific competition is alleviated with increasing phenotypic distance between individuals. If the molecular forms differ in their antipredator response leading to increased habitat segregation and reduce resources overlap, then divergent selection will become stronger [2]. Predator pressure in rice fields is higher than in temporary puddles [28-30]. Accordingly, we hypothesized that predation is the key factor that shapes the segregation pattern in the occupation of larval sites, hence dictates the micro-geographic distribution of the forms. The following predictions were tested: (i) under predator pressure, developmental success of the M form larvae will increase whereas that of the S form will decrease in both habitats. (ii) in absence of predator pressure, developmental success of the S form larvae will increase whereas that of the M form will decrease in both habitats. In a field experiment using transplantation of first instar larvae, we estimated development capacity of the molecular forms in both temporary (puddle) and permanent (rice fields) larval sites in the presence versus absence of predation. Here we present evidence that the molecular forms have adapted to different types of larval sites (habitats) and discuss the evolutionary implications for speciation in *An. gambiae*.

## Results

### Overall developmental success

**Figure 1:**
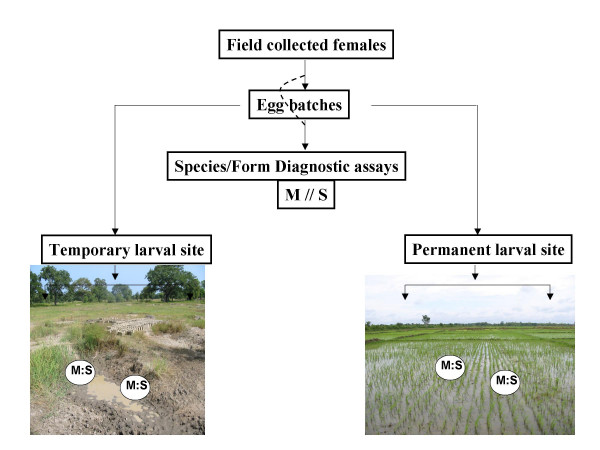
Diagrammatic illustration of the experimental design.

In a field experiment using transplantation of first instar larvae (Fig. 1), we evaluated the fitness of the molecular forms, measured as the emergence success of adults and their developmental time, in rice fields and puddles with or without predation effect. A total of 19 pairs of cages (38 cages in total) were set in nine puddles and in ten rice paddies during the rainy season of 2004 (May–October 2004). Five cages were damaged by floods caused by heavy rains or by children before all adults were collected. These cages were excluded from all data sets. In total, 3,710 adults were collected from 33 transplantation cages and species identification was performed on 3,629 adults (81 specimens were lost before identification).

**Figure 2:**
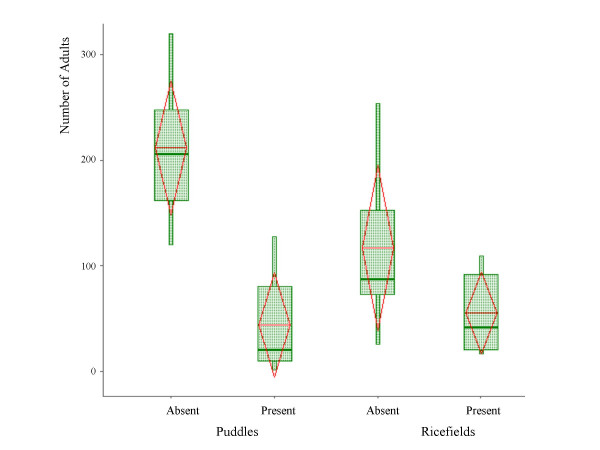
Larval developmental success (measured as the number of adults per cage) of the molecular forms in presence and absence of predators in puddles and rice fields. The box extends between the 25^th ^and the 75^th ^percentile (across the inter quartile range – IQR) and the median is denoted by a thick line. The whiskers extend up to the most extreme value, up to 1.5 times the IQR and values located over 2 IQR from the median are shown. The triangles extend from the mean (base) to 1 SD (tip).

Emergence success was over three fold higher in predator free cages than in cages with predators (164.8 adults/cage and 49.6 adults/cage respectively) reflecting a strong predator effect on larval success. Puddles were significantly more productive than rice fields in absence of predation (212 adults/cage and 117.56 adults/cage respectively; df = 1, *P *= 0.012, Fig. 2), but no difference between habitat was detected in presence of predators (df = 1, *P *= 0.635, Fig. 2). Emergence success of males and females was similar (overall 1,868 females and 1,844 males; df = 1, *P *= 0.645, χ^2 ^test).

### Predator composition and abundance in rice fields and puddles

**Figure 3:**
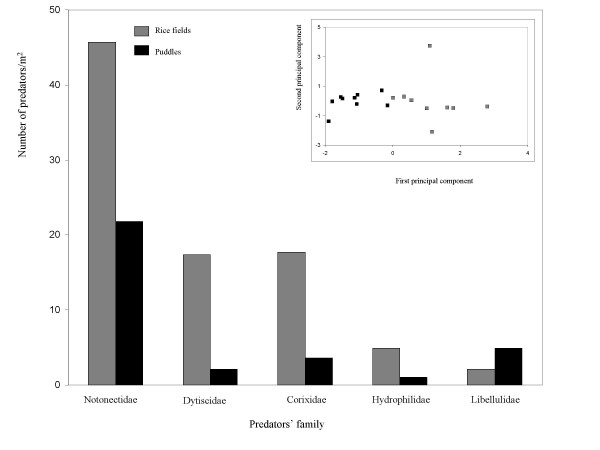
Predator composition in rice fields (empty squares) and puddles (filled squares). Bars denote mean density in 10 samples of rice fields and 9 samples of puddles of each predator. Stars above bars denote significant difference in a single test. Inset shows clustering of samples based on their predator composition using Principal Component (PC) analysis. Coordinates are the first (horizontal) and second principal components. The first PC represented overall predator abundance because its eigenvector's loadings were positive and similar in magnitude (except for the negative loading of the dragonfly; not shown). It alone accounted for 38% of the total variation and together with the second PC, 64% of the total variation was captured.

Predators were sampled in all larval sites where experimental cages were transplanted (see Materials and Methods) and their numbers were subjected to a MANOVA test to evaluate difference in their abundance and composition between habitats. Five predatory taxa were identified in this series of experiments as Hemiptera: Notonectidae, *Anisops *sp. and *Anithares *sp. (backswimmer), Hemiptera: Corixidae, *Micronecta *sp. (water boatman), Odonata: Libellulidae, *Tramea *sp. (dragonfly), and two adult beetles, Coleoptera: Hydrophilidae, *Berosus *sp. and Coleoptera: Dytiscidae, *Laccophilus *sp. The results showed that the number of predators was higher in rice fields than in puddles (Fig. 3, F = 8.78, df1 = 5, df2 = 12, *P *= 0.0011). Backswimmers were the most abundant predators in both rice field and puddles with a mean collection of 45.7 and 21.8 predators/m^2 ^respectively. A significant difference in predator composition between habitats was found using a Principal Component analysis (Fig. 3).

### Developmental success of the molecular forms

Stratified contingency table analyses by habitat showed that predation increased the developmental success of the M form (*P *= 0.0004, df = 1, Table 1). In separate analysis for each habitat this pattern was significant in puddles (*P *= 0.0006, Table 1), but although the same trend was found in rice fields, the difference was not significant (*P *> 0.124, Table 1). Without predation, the molecular forms exhibited a higher developmental success each in its natural habitat (*P *< 0.0001, Table 1) in an apparent contrast to our previous study [25] (see Discussion). A logistic regression analysis accommodating variation among cages with habitats showed a significant effect of both predation and habitat on the success of the forms (*P *< 0.0001 and *P *= 0.0019 respectively, Table 2). Consistent with the contingency table analyses (above), predation increased the developmental success of larvae of the M form in both habitats, and each form displayed a higher developmental success in its typical habitat. No predation*habitat interaction effect was detected, hence the effect of predation on the emergence success of the forms was similar across habitats (*P *= 0.13, Table 2). Mosquito sex and its interactions with habitat (*P *= 0.69) or with predation (*P *= 0.93) were not significant suggesting that predators feed upon males and females equally across habitats (data not shown). The effect of individual predator species on developmental success of the forms was evaluated using logistic analysis (Table 3). Higher density of predators belonging to Notonectidae and Dytiscidae families increased the relative success of the M form (*P *< 0.0001 and *P *= 0.02 respectively Table 3). Notably, higher density of Libellulidae and Hydrophilidae specimens appeared to decrease the relative success of the M form, but their effects were not significant.

**Table 1:** Overall effect of predation on relative developmental success of the molecular forms in puddles and rice fields.

Habitat	Predator	M form	S form	χ^2^/P
Puddles	Absent	46.3% (857) a	53.7% (993)	11.65/0.0006
Puddles	Present	56.2% (199)	43.8% (155)	
Rice fields	Absent	55.1% (576)	44.9% (470)	2.35/0.124
Rice fields	Present	59.6% (226)	40.4% (153)	
Total (Pooled data)	Absent	49.5% (1433)	50.5% (1463)	16.9/0.0001
	Present	58% (425)	42% (308)	12.4/0.0004b

a () number of adults emerged

b stratified analysis of form by predation controlling for habitat using Cochran-Mantel-Haenszel Test.

**Table 2:** Emergence success of the molecular forms in different habitats using a logistic regression accommodating cages variation

Source	Df	*P*	Estimate [ln(M/S)]
Intercept	1	0.84	-0.0232
Predator	1	<0.0001	+0.258
Habitat	1	0.019	+0.272a
Predator*Habitat	1	0.13	+0.08b
GroupCage (Habitat)	17	<0.0001	NA
Likelihood Ratio	11	<0.0001	NA

a: puddle over rice

b: predator over no predator in puddle/predator over no predator in rice

**Table 3:** Effect of individual predator on the relative success of the forms: logistic analysis

Source	Df	P	Estimate [ln(M/S)]
Intercept	1	0.013	-0.263
Habitat	1	0.05	+0.206*
GroupCage (Habitat)	17	<0.0001	NA
BackSwimmer	1	<0.0001	+0.202
Dragonfly	1	0.14	-0.33
Pinkwbug	1	0.01	+0.305
Blackwbug	1	0.09	-0.323
FD	1	0.65	+0.05
Likelihood ratio	8	<0.0001	NA**

*: puddle over rice, ** Not Applicable

### Developmental time

**Figure 4:**
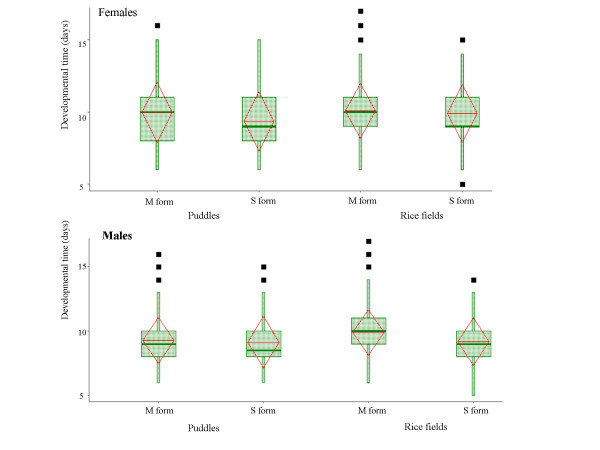
Distribution of developmental time of the molecular forms in each habitat (by sex). Developmental Time was measured from transplantation of first instar to adult. The box extends between the 25^th ^and the 75^th ^percentile (across the inter quartile range – IQR) and the median is denoted by a thick line. The whiskers extend up to the most extreme value, up to 1.5 times the IQR and values located over 2 IQR from the median are shown. The triangles extend from the mean (base) to 1 SD (tip).

Overall, the developmental time was shorter in cages with predators than without predators (8.74 vs. 9.55 days; F = 110.02, df = 12, *P *< 0.0001), probably reflecting the diminished number of larvae surviving predation over time. Therefore, we used predator free cages to compare differences in developmental time between the molecular forms. As expected, males developed slightly faster than females in both habitats (P < 0.0001, Table 4; Fig. 4) and both sexes developed faster in puddles (P < 0.0001, Table 4; Fig. 4) as previously reported [25]. Importantly, the S form developed faster than the M form across habitats (P < 0.0001, Table 4, Fig. 4).

**Table 4:** ANOVA for the developmental time of males and females of the molecular forms in different habitats

Source	Df	*P*
Form	1	<0.0001
Habitat	1	<0.0001
Form*Habitat	1	0.6285
Sex	1	<0.0001
Form*Sex	1	0.2246
Sex*Habitat	1	0.7615
Form*Sex*Habitat	1	<0.0001
Cage (Habitat)	16	<0.0001

## Discussion

The ultimate objective of this study was to identify ecological differences between the molecular forms of *An. gambiae *that might reflect the evolutionary forces producing their divergence. Prompted by the failure to detect adaptive difference between forms in predator free settings [25], we evaluated the role of larval predators in mediating divergent selection that explains the sharp habitat segregation exhibited by the forms with respect to rice cultivation areas vs. surrounding savanna. We found significant difference in the ability of the molecular forms to exploit the different larval sites conditioned on the presence of predators. In absence of predation, the molecular forms outcompeted each other in their natural habitats (S form being better in puddles and M form being better in rice fields) however, the developmental success of the M form was higher than that of the S form in both habitats under predator pressure. Consistent with the previous study which emphasized the role of competition at least in puddles [25], our results suggest that both competition and predation shape the pattern of habitat segregation exhibited by the molecular forms; the S form outcompeting the M form in temporary low predation larval sites, and the M form being better in permanent larval sites with high predation. The implications of these results extend beyond the geographical and environmental segregation between the molecular forms into the processes involved in their divergence.

The ecological and genetic processes of species formation are key to understanding how biological diversity is generated. The molecular forms of *An. gambiae *have been extensively studied for the last decade, and yet the exact mechanisms of their divergence are obscure. So far, few experimental studies have been designed to look at phenotypic traits between the forms and all failed to find consistent differences between them [31,32]. This is the first empirical evidence for specific adaptive differences between the molecular forms and it stresses the role of larval predation as one of the mechanisms contributing to their divergence [7,8,15,17,19,20,22]. Dobzhansky believed that speciation in *Drosophila *proceeds mainly through evolving physiological complexes which are successful each in its environment [33] and Mayr recognized that many of the accumulative genetic differences between populations, particularly those affecting physiological and ecological characters, are potential isolating mechanisms [34]. Recently, several studies have provided evidence in support of divergent selection as evolutionary force driving speciation in different species. A manipulative field experiment using enclosures showed that both competition and predation served as mechanisms of adaptive radiation in *Timema *stick insects [6,35] and that predation promotes premating isolation [36] in walking-sticks.

Among the various natural ecologic forces controlling vector populations, predation seems to be the most important. Predator species may vary across a prey species' range [27] but we emphasize that predators manipulated in our experiment probably represent the key predators of *An. gambiae *since independent studies on larval predators of *An. gambiae *in East Africa [26,27] have identified key larval predators that match those found in our study. A generalization of our results depends on how much predators in *An. gambiae *larval sites rely on this mosquito rather than on other prey and to what extent medium and large larval sites used in this study account for *An. gambiae *total productivity? Predators certainly prey on a variety of small aquatic invertebrates but not much is known about the diet of these predators in larval sites of *An. gambiae*. Service [27] could not identify alternative prey in *An. gambiae *larval site in Kenya, therefore concluded that predation was mainly limited to *An. gambiae*. We have found larvae of other mosquito species in the same sites only on few occasions suggesting that *An. gambiae *was probably the main prey that predators could feed on. Additionally, medium and large larval sites as used in this experiment probably represent the typical conditions *An. gambiae *grows in as studies on larval sites predators revealed. Mutuku and collaborators have found that larger sites such as quarries produced more adult mosquitoes and accounted for about 85% of *An. gambiae *adults [37]. Small larval sites, such as hoof prints promote larval development but fail usually to produce pupae. High probability of desiccation of the small larval sites was an important factor accounting for their failure to support complete development.

Recent study has revealed cannibalism and predation among larvae of *An. gambiae *complex [38] but we suspect such predation between the molecular forms of *An. gambiae *to be minor since our results indicated that the emergence success of the forms was over three fold higher in predator free cages than in predator present cages. Further our experiment consisted of transplanting L1s so variation in larval size was minimized. The mechanism conferring predation avoidance in the M form is not known. Juliano and Graves have shown that the co-evolution of predator and its prey can rapidly select for divergence in prey behaviour [39]. Aquatic animals use chemical cues for behavioural decision making relating to foraging, reproduction and the assessment of predation risk [39,40]. Defensive responses of prey to predators include increased use of refuge [41], reduced foraging and change in rate of movement [42]. The S form larvae develop faster than those of the M form in both habitats probably as a mechanism to avoid larval site desiccation. This likely requires more active foraging that increases exposure to predators and outweighs the benefit of shorter development time. Recently a molecular assay to detect predation on *An. gambiae *larval stages in the gut of different predators was developed. This assay can be used to follow up on the dynamic and the structure of both predators and the molecular forms of *An. gambiae *in different ecological environments.

Divergence between populations of species inhabiting freshwater bodies based primarily on the length of hydroperiod has been proposed for a number of species. Rice fields are relatively permanent larval sites whereas rain puddles, especially early in rainy season represent extremely ephemeral bodies of water. The temporal fluctuation in the availability of temporary versus permanent larval sites explains the seasonal change in the frequencies of the forms, whereby the M dominates in the dry season when only permanent larval sites are available and the S in the rainy season as well as the corresponding latitudinal cline [8,24,43]. In locations where predation pressure is intermediate, both forms may be similarly successful, which might explain the high rate of cohabitation in an area where the forms were sympatric [44]. We propose that similar differences are found between *An. arabiensis *and *An. gambiae *in East Africa where the former fills the niche of the M form in West Africa [45]. Our results highlight the role of larval rather than adult adaptations as the life stage that drives this turnover.

Natural selection should favour females that oviposit in sites providing the best available conditions for their progeny. Ovipositing mosquito females challenge in their life time desiccation and predation. Our results showed that rice fields and puddles contrast significantly in the number of aquatic predators and we assume that in the prospect of ovipositing M and S females, these larval sites are different. Because the prospects of larvae in different habitats depend on their molecular form, it is expected that female's oviposition site selection has been under selection accordingly [46]. The observed geographical and ecological segregation between forms is probably augmented by female choice of oviposition site.

## Conclusion

Uncovering the ecological and genetic mechanisms of species differentiation is a key to understanding how biological diversity is generated. Many studies are ongoing to better understand the process of speciation within *An. gambiae*. While previous studies failed to provide evidence in support of this differentiation, recent data are emerging in support of M and S distinctions. The results of the present study are consistent with this idea. We found consistent differences in the ability of the forms to exploit different larval sites and identified the ecological agents involved. Both larval predation and inter-form competition may commonly serve as mechanism of divergent selection [6].

## Methods

### Study areas

The rice fields surrounding the village of Bama, located 30 km from Bobo Dioulasso, Burkina Faso, were selected as a typical M-form environment. The district of Kuinima on the periphery of Bobo-Dioulasso was selected as a typical S-form environment. A detail description of these areas is found in [25]. In each area, only one form predominates (>90%) during the rainy season [47].

### Predator identification

In each habitat, at least five larval sites were sampled prior to the transplantation experiment to identify the main predators in these sites. After sighting of *An. gambiae *larvae, a cylinder (70 cm diameter 80 cm height) was quickly pushed into the mud to contain the water column over a constant sampling surface. All visible macro-invertebrates were collected and the bulk of water and upper layers of mud/rocks were carefully inspected in white pans to find hidden organisms. Invertebrates were brought to the laboratory of IRSS/Centre Muraz to assess their role as predators. They were sorted under a dissecting scope and one specimen of every "type" of invertebrate was placed in a pan (30 cm diameter, 10 cm deep) filled with 0.5 litre of deionised water, with 10 first or second instar larvae and 10 third or fourth instar larvae of *An. gambiae*. One control pan with the same larval composition, but without a predator was included in each set of experiments. Surviving larvae after 24 hrs were counted and observed predation events were recorded. Three to five replicate experiments were conducted with every predator type. If predation was observed in at least 2/3 of the experiments with the same taxon, it was considered a predator. Five predatory taxa were identified in this series of experiments as Hemiptera: Notonectidae, *Anisops *sp and *Anithares *sp. (backswimmer), Hemiptera: Corixidae, *Micronecta *sp. (water boatman), Odonata: Libellulidae, *Tramea *sp. (dragonfly), and two adult beetles, Coleoptera: Hydrophilidae, *Berosus *sp. and Coleoptera: Dytiscidae, *Laccophilus *sp. We believe that these five taxa represent the key larval predators in our study area.

### The transplantation cages

Cylindrical cages (diameter = 70 cm, height = 80 cm) made of metal frame were fitted from the bottom to the middle with a cloth to contain the larvae but allow exchange of water, small particles, and microorganisms. The cloth's pore was elliptic with mean length of 0.12 mm (SD = 0.04) and mean width of 0.08 mm (SD = 0.026). From the middle to the top, the cage was covered with a regular mosquito net to prevent adult mosquitoes and other invertebrates from entering or exiting the cage. The upper cloth was fitted with a "sleeve" through which adult mosquitoes were aspirated from the cage. The cage was secured to the ground using three stakes.

### Larvae transplantation and adult collection

Gravid and bloodfed *An. gambiae *females were collected indoors in Bama and Kuinima and provided with 5% sugar water for 48–96 hours in the laboratory. At that time, they were individually transferred into ovipostion cups. After they laid eggs, the females were preserved in 85% ethanol and their molecular form was determined by PCR performed on a single leg [11].

Batches of 200 one day old larvae, representing 2–3 families of each molecular form, were counted, placed in 50 ml plastic bag, and quickly transferred into the field (Fig. 1). In each larval site, defined as a body of water where larvae of *An. gambiae *could be detected, two cages were placed approximately 1 m apart (pairs of cages in the same habitat were at least 10 m apart). A total of 400 larvae of both molecular forms (200 M: 200 S) were placed into each cage and predators found *in situ*, were added into one cage whereas the other remained predator free. To collect predators, a bottomless cage was inserted into the mud and secured, the bulk of the water and top mud layer were removed into pans and predators were collected, identified and counted. Then the cloth was inserted into the frame and secured. To complete the cage setup, previously collected and dried mud (from the same area) was introduced and formed a shallow and narrow (approximately 5 cm wide) edge covering approximately 1/3 of the periphery of the cage to serve as refuge against predators. When water levels stabilized, the larvae were introduced slowly into the cage. Half the number of each type of predator collected *in situ *were introduced into the cage 15 minutes after the larvae were added (remaining predators were preserved). No more than five notonectids were added into any cage even if more than ten were collected because earlier experiments indicated that no adults were produced in cages with higher numbers of notonectids. All the cages were secured with stakes.

After setup, cages were checked daily and emerged adults collected until no pupae, larvae, or adults were observed for two consecutive days. Emerged adults were counted and preserved in 85% ethanol 24 hours after emergence. Their molecular form was determined by a PCR-RFLP assay [48].

### Data Analyses

Developmental success of the molecular forms in transplantation cages was measured by the total number of adults that emerged from each cage and by larval developmental time. The total number of adults of each form in each habitat with- and without predation was analyzed using contingency table Chi Square test to examine overall trends. Logistic regression analyses were used to accommodate variation among cages to test the effects of the habitat, predation, sex, cage set (consisting pair of cages set approximately 1 m apart in the same larval site), and their interactions on the probability to produce M vs. S adult. An analysis of variance (ANOVA or MANOVA) was performed to compare the larval developmental time (treated as a continuous variable) and the predator density in the different habitats. Principal Component analysis was used to evaluate the difference between habitats in their predator profile. Statistical analyses were performed using SAS [49].

## Authors' contributions

The work presented here was carried out in collaboration between all authors. DA, LT and DKR designed the study. DA carried out the field and laboratory work, participated in the analysis of data, interpreted the results and wrote the paper. DKR carried out the field and laboratory work and revised the manuscript. HK and CJ carried out the laboratory work and revised the manuscript. WL and CL identified the predators' specimens and revised the manuscript. LT analysed the data, interpreted the results and revised the manuscript. All authors have read and approved the final manuscript.
